# Effect and Mechanisms of Yiqi Jianpi Xiaoyu Prescription on Kidney Fibrosis on the Basis of Metabolomics and Experimental Validation

**DOI:** 10.1002/fsn3.71123

**Published:** 2025-10-29

**Authors:** Keda Lu, Liqing Ye, Wenze Jiang, Tianyang Cheng, Hong Xia, Peipei Zhang, Bingbing Zhang

**Affiliations:** ^1^ Department of Nephrology Hangzhou Third Hospital Affiliated to Zhejiang Chinese Medical University Hangzhou China; ^2^ Department of Nephrology The First Affiliated Hospital of Zhejiang Chinese Medical University Hangzhou China; ^3^ The Third School of Clinical Medicine (School of Rehabilitation Medicine), Zhejiang Chinese Medical University Hangzhou China; ^4^ School of Pharmaceutical Sciences Zhejiang Chinese Medical University Hangzhou China

**Keywords:** adenosine, autophagy, high performance liquid chromatography, kidney fibrosis, traditional Chinese medicine, Yiqi Jianpi Xiaoyu prescription

## Abstract

The Yiqi Jianpi Xiaoyu prescription (YQJPXY) exhibits ameliorative effects on kidney fibrosis, but the underlying mechanism related to metabolism remains insufficient. We aimed to explore the antifibrotic effects of YQJPXY on kidney fibrosis and its underlying mechanism using serum metabolomics, biochemical analyses, and experimental analyses. We established a unilateral ureteral obstruction (UUO) kidney fibrosis mouse model, dividing mice into sham, model, YQJPXY‐treated (7.28, 14.56, 29.12 g/kg/day), and Losartan groups. Renal function, histopathology, and fibrosis markers (FN, Col IV, and α‐SMA) were analyzed. Serum metabolomic (UHPLC‐Q‐TOF/MS) identified key metabolites. TEM, qRT‐PCR, WB, ELISA, and immunohistochemical validation confirmed the Adenosine mechanism in promoting autophagy through the A2BR/cAMP/AMPK pathway in the process of YQJPXY against kidney fibrosis. TGF‐β‐induced HK‐2 cell fibrosis with autophagy inhibitor (CQ), A2BR inhibitor (PSB1115), and AMPK inhibitor (Dorsomorphin) explored the mechanism. YQJPXY improved kidney function and fibrosis. Metabolomics identified five important metabolites in kidney fibrosis: Adenosine monophosphate, Adenosine, Adenine, Inosine, and Hypoxanthine. Among these, Adenosine, which attenuated fibrosis via A2BR/cAMP/AMPK‐mediated autophagy, was inhibited by CQ, PSB1115, and Dorsomorphin. YQJPXY's antifibrotic mechanism involves adenosine‐activated A2BR/cAMP/AMPK autophagy pathways.

## Graphical abstract

Our results demonstrate that YQJPXY alleviates fibrosis by regulating key metabolites, including adenosine, adenine, and inosine, and activates autophagy through the A2BR/cAMP/AMPK pathway.

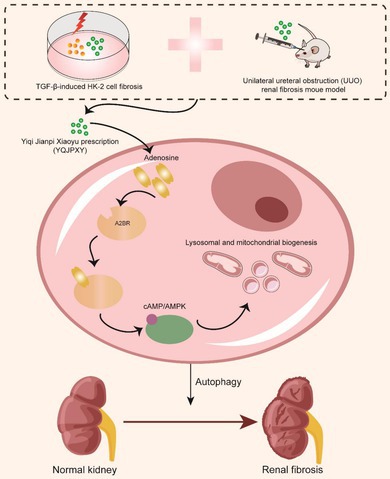

## 1 Introduction

The prevalence of chronic kidney disease (CKD) has been rising recently and has affected 119.5 million people in China (Zhang et al. 2012) and 697 million people worldwide (Global, Regional, and National Burden of Chronic Kidney Disease, 1990–2017: A Systematic Analysis for the Global Burden of Disease Study 2017 2020). CKD is associated with a higher cardiovascular risk and a significant proportion of patients may eventually advance to end‐stage renal disease (ESRD), which requires dialysis or renal transplantation (Evans et al. 2022). The cost of CKD was estimated at $49 billion per year, creating a significant economic burden (Lv and Zhang 2019). However, patients with CKD are often undiagnosed because of its asymptomatic characteristics in the early stages (Ammirati 2020). Hence, investigating the effective biomarkers in CKD may benefit the early diagnosis and underlying mechanism exploration for CKD.

Development of fibrosis is the basis and ultimate pathological feature of CKD. The mechanism of kidney fibrosis is extremely complex, involving the interactions of multiple cells, proteins, and signaling pathways (Panizo et al. 2021). Kidney fibrosis is driven by abnormal accumulation of extracellular matrix (ECM) components, including collagen, fibronectin (FN), and alpha‐smooth muscle actin (α‐SMA). In this process, the fibroblasts are differentiated into myofibroblasts and induce scarring (Miguel et al. 2025). Considering that kidney fibrosis is the final common outcome of a wide variety of CKD, it is possible that understanding the underlying mechanism of kidney fibrosis could offer effective insights for the development of therapeutic interventions.

Traditional Chinese medicine (TCM) has attracted great attention in the treatment of kidney disease because of its potential clinical advantages (Shen et al. 2024; Wu et al. 2024). The Yiqi Jianpi Xiaoyu (YQJPXY) formula is first recorded in Correction in the Errors of Medical Works and is famous for tonifying qi, strengthening the spleen, removing blood stasis, and purging dampness turbidity (Xia, Zhang, et al. 2021). YQJPXY is comprised of nine herbs, including Radix Astragali (Huangqi), Radix Cyathulae (Chuanniuxi), Semen Persicae (Taoren), Lumbricus (Dilong), Chinese rhubarb (Zhijun), plantain herb (Cheqiancao), Radix Codonopsis (Dangshen), Poria (Fuling), and white Atractylodes rhizome (Baizhu), which exhibit efficacy in promoting blood circulation, removing blood stasis, and clearing away turbid toxins. The YQJPXY formula has been included in nosocomial preparations for treatment in kidney diseases like acute kidney injury (K. Lu, et al. 2011; Lu et al. 2016). Xia et al. emphasized that YQJPXY improves myophagism in nephrectomized rats through the IGF‐1/PI3K/Akt pathway (Xia, Zhang, et al. 2021). The nine components of the YQJPXY formula also indicate an effective improvement in kidney fibrosis and kidney disease. Huangqi and its active ingredients have been demonstrated to exert an antifibrotic effect through regulating TLR4/NF‐κB mediated reduced neutrophil extracellular traps NET in chronic nephritis (Cao et al. 2025). Zhijun demonstrates efficacy in reducing proteinuria, lowering blood sugar levels, and improving kidney function (S. Fu et al. 2022). Cheqiancao can effectively decrease the levels of fibrin and collagen by inhibition of inflammatory factors and NF‐κB in diabetic kidney disease (Lan et al. 2024). The regulatory effects of Dangshen on immune regulation and liver and kidney damage have also been emphasized (Shi et al. 2024). Fuling exhibits a diuretic effect and improves kidney diseases (Guo et al. 2025). Baizhu and its active ingredients improve the inflammatory response and glomerular lesions in the immunoglobulin A nephropathy model mice (Ishii et al. 2020). The antifibrotic effects of Chuanniuxi and Taoren in kidney disease remain obscure, but their anti‐hepatic fibrosis effects have been reported (Huang et al. 2025; Meng et al. 2019). These pieces of evidence collectively demonstrate the advantages and potential of the YQJPXY formula and its main components in the treatment of kidney‐related diseases. Nevertheless, the specific mechanism of YQJPXY in decelerating kidney fibrosis remains insufficient.

In the procession of CKD to ESRD, 28%–54% of the patients were accompanied by varying degrees of metabolic disorders and malnutrition (Carrero et al. 2018). Studies have pointed out that changes in metabolites indicate kidney fibrosis progression and are regulated by relevant genes (Chen et al. 2024, 2023; Gao et al. 2021). For example, WWP2 (a multifunctional E3 ubiquitin–protein ligase) is reported to be up‐regulated in fibrotic kidney tubule interstitium and controls mitochondrial respiration through inhibition of PGC‐1α to promote kidney fibrosis (Chen et al. 2024). Monoacylglycerol lipase (MAGL) is significantly reduced in CKD, consistent with lipid accumulation and kidney fibrosis (Zhou et al. 2024). Specific transgene of MAGL in tubular cells can inhibit lipid‐mediated toxicity and mitigate fibrogenesis. Importantly, the regulatory roles of various TCMs (Fang et al. 2016; Gao et al. 2021; Wang et al. 2023; Zhao et al. 2023), including YQJPXY (Ding et al. 2022; Fu et al. 2024; Lu, et al. 2011; Xia, Ye, et al. 2021; Zhuang et al. 2023), have been proposed in kidney fibrosis, with mechanisms including amino acid metabolism, energy metabolism, and microbial regulation. These studies prove that changes in metabolic levels are associated with kidney fibrosis, and YQJPXY can inhibit kidney fibrosis by regulating metabolic levels. Nevertheless, important metabolites and potential mechanisms in the therapy of YQJPXY against kidney fibrosis remain deficient.

Metabolomics is a novel method to screen significant biomarkers in disease and is beneficial to explore the multiple targets and metabolic pathways of TCM (Liu et al. 2024). In this study, we established a rat model of kidney fibrosis by unilateral ureteral obstruction (UUO) and investigated the important metabolites and the underlying mechanism of YQJPXY action in kidney fibrosis by metabolomic analysis. Our findings provide an experimental basis for the pharmacological analysis of other TCM formulations.

## 2 Methods

### 2.1 YQJPXY Preparation

In this study, YQJPXY comprised Astragali Radix (30 g), Radix Cyathulae (12 g), Semen Persicae (12 g), Lumbricus (12 g), Chinese rhubarb (10 g), plantain herb (20 g), Radix Codonopsis (15 g), Poria (15 g), and white Atractylodes rhizome (15 g). All the above materials were provided by Hangzhou Huadong Pharmaceutical Co. Ltd. (Hangzhou, China), and the decocting method referred to a previously published work (Xia, Zhang, et al. 2021).

### 2.2 Animals and Treatment

In total, 48 6–8‐week C57BL/6J mice (6–8 weeks old, 20–25 g weight) were provided by the experimental animal center in Zhejiang Chinese Medical University. All mice were kept in captivity in 12/12‐h light/dark cycles with a temperature of 25°C and were fed and watered freely. All the animal experiments complied with ARRIVE guidelines.

After adoptive culture for 1 week, all the mice were randomly grouped into six groups (*n* = 8): (1) Sham; (2) Model; (3) Low‐dose YQJPXY group; (4) Middle‐dose YQJPXY group; (5) High‐dose YQJPXY group; and (6) Losartan (positive control, commonly used in UUO mouse model). The kidney fibrosis mouse model was established by UUO as a previous publication (Chen et al. 2023). Mice in the model group were anesthetized with pentobarbital sodium by intraperitoneal injection (50 mg/kg), exposed to the proximal end of the left ureter, and ligated with 6–0 silk. Mice in the sham group were operated on using the above method without ligated ureter. On the second day after surgery, the mice in low‐, middle‐, and high‐dose YQJPXY groups were given YQJPXY intragastric administration for 14 days, with 7.28, 14.56, and 29.12 g/kg/day, respectively. The dose of YQJPXY was calculated according to the clinical dose and body surface method. In the Losartan group, 10 mg/kg of Losartan was administered intragastrically once a week as reported (Lee et al. 2013; Zou et al. 2022). At the end of all experiments, all mice were euthanized after receiving isoflurane inhalation anesthesia for cervical dislocation. The blood and kidney tissues of each mouse were collected.

### 2.3 YQJPXY Serum Preparation

Retroorbital blood samples were collected from the sham and middle‐dose mice. After centrifugation at 3000 r/min for 15 min, the serum was inactivated at 56°C for 30 min, filtered (0.22 μm) to remove bacteria, and stored at −80°C.

### 2.4 Cell Culture and Treatment

Human kidney proximal tubular epithelial cells HK‐2 (CL‐0109, Prexel) were incubated in DMEM/F12 containing 10% FBS and 1% penicillin–streptomycin at 37°C and 5% CO_2_. The cells were treated with 10 ng/mL TGF‐β for 48 h to establish a fibrotic cell model. Adenosine (SD7162‐25 mg, Beyotime), autophagy inhibitor chloroquine (CQ, 53755ES60, Yeasen Biotechnology), A2BR inhibitor PSB1115 (GC44733, GlpBio), and AMPK inhibitor Dorsomorphin (ab120843‐10 mg, Beyotime) were added to the HK‐2 cells for the underlying mechanism investigation.

### 2.5 Detection of Markers of Kidney Injury and Dysfunction by Automatic Analyzer

The markers of kidney injury and dysfunction of mice were detected after treatment for 14 days. Urine was collected after fasting for 12 h, and the clinical markers of urine kidney injury and dysfunction, such as urine urea nitrogen (UN), urinary creatinine (UCR), and urinary albumin excretion rates (UAER), were measured using automated biochemical analyzers to assess kidney function in mice.

### 2.6 HE, Masson Staining, and PAS Staining

Mouse kidneys were fixed in 4% paraformaldehyde (P0099‐100 mL, Beyotime) for 24 h and embedded in paraffin. Paraffin was cut into sections using a paraffin microtome (RM2235, Lecia). The sections were deparaffinized with xylene, dehydrated through ethanol, and stained with hematoxylin–eosin (HE) (C0105S, Beyotime), Masson (G1340, Solarbio), and PAS staining.

### 2.7 Immunohistochemistry

Kidney tissues were fixed in 4% methanol, paraffin‐embedded, and sliced at 4 μm. The sections were incubated with normal goat serum for 15 min, followed by incubation with anti‐FN (1:2000, ab268020, abcam), anti‐Collagen Type IV (Col IV) (1:400, ab6586, abcam), anti‐α‐SMA (1:1000; ab124964, Abcam), Beclin‐1 (1:500, ab62557, abcam), LC3II (1:200, AF4650, Affinity), and p62 (1:500, ab155686, abcam). Subsequently, the sections were cultured with a goat anti‐rabbit IgG H&L (HRP) secondary antibody (1:1000; #ab6721, Abcam) for 15 min. Development was realized using DAB (P0202, Beyotime). The sections were counterstained with hematoxylin for 3 min, rinsed with running water, and dehydrated with xylene and ethanol.

### 2.8 CCK‐8

A CCK‐8 kit (C0037, Beyotime) was employed to detect cell viability. In brief, HK‐2 cells were collected and seeded in 96‐well plates (100 μL, 2000 cells/well) and cultured for 24 h. After culturing for 24 h, 10 μL CCK‐8 reaction liquid was added. Optical density was detected at 450 nm using a DR‐3518G microplate reader (Hiwell Diatek).

### 2.9 Transmission Electron Microscopy (TEM)

TEM was used for the detection of autophagy lysosomes.

### 2.10 cAMP Detection

ELISA (EU2574, FineTest) was used for the detection of cAMP levels. The cells were centrifuged to obtain cell supernatant. The levels of cAMP in cell supernatant were detected using corresponding HRP Streptavidin (SABC) and TMB according to the manufacturer's instructions. Optical density was detected at 450 nm.

### 2.11 qRT‐PCR

Total RNA was extracted from kidney tissue using a TRIzol reagent (15596018; Invitrogen, Carlsbad, CA, USA) following the manufacturer's instructions. cDNA synthesis was conducted using FastKing‐RT SuperMix (KR118‐02; Tiangen). qRT‐PCR analysis was performed using SYBR Green PCR Master Mix (A4004; Thermo Fisher Scientific, MA, USA). Gene expression was computed using the $2^{-\Delta \Delta C_{t}}$ method with GAPDH as the internal control. The primers used in this study are listed in Table 1.

**TABLE 1:** The primers used in the qRT‐PCR.

Gene name	Primer sequences (5′–3′)
Beclin‐1	Forward: 5′‐CAGTACCAGCGGGAGTATAGTGA‐3′
	Reverse: 5′‐TGTGGAAGGTGGCATTGAAGA‐3′
LC3	Forward: 5′‐TACTGTGGCGGGAATTGCTC3′
	Reverse: 5′‐ACCCCAAGGCCATTGGTTAC‐3′
p62	Forward: 5′‐ACCCCCGTGTGAAGCTTATG‐3′
	Reverse: 5′‐CACAGATACCCCACGACCAC‐3′
A1	Forward: 5′‐GTGGCCTGGCAGATACTCAG‐3′
	Reverse: 5′‐TAGAAGGGGGAGGGGTAAGC‐3′
A2A	Forward: 5′‐ACTGCAGAAGGAAGTCCACG‐3′
	Reverse: 5′‐ACGTGGAGCAGAAGAAGGTG‐3′
A2B	Forward: 5′‐GTTTGGTCACTGGGACACGA‐3′
	Reverse: 5′‐CCCCAGGAACGGAGTCAATC‐3′
A3	Forward: 5′‐TCCAAAGGAACCAGAGCAGC‐3′
	Reverse: 5′‐CACACATTGCAGCATCCACC‐3′
GAPDH	Forward: 5′‐TGGATTTGGACGCATTGGTC‐3′
	Reverse: 5′‐TTTGCACTGGTACGTGTTGAT‐3′

### 2.12 Western Blotting (WB)

WB was used for the detection of protein levels of fibrosis proteins (FN, Col IV, and α‐SMA), autophagy‐related proteins (Beclin‐1, LC3II/LC3I, and p62), adenosine receptor proteins (A1R, A2AR, A2BR, and A3R), and cAMP/AMPK pathway proteins (AMPK and p‐AMPK). Total protein was isolated using RIPA lysis buffer (P0013B; Beyotime), and then the protein concentration was quantified using a bicinchoninic acid (BCA) kit (PC0020; Solarbio). The membranes were incubated with primary antibodies at 4°C overnight, followed by incubation with goat anti‐rabbit IgG H&L (HRP) (1:2000; ab6721, Abcam) secondary antibody for 1 h. GAPDH served as the internal control. Primary antibodies used in this assay were as follows: anti‐FN antibody (1:1000, ab268020, Abcam), anti‐Col IV antibody (1:1000, ab6586, Abcam), anti‐α‐SMA antibody (1:1000, ab124964, Abcam), anti‐Beclin‐1 antibody (1:1000, ab302669, Abcam), anti‐LC3II/LC3I antibody (1:2000, TA5402, Abmart), anti‐p62 antibody (1:2000, 5114, Cell Signaling), anti‐A1R antibody (1:1000, ab82477, Abcam), anti‐A2AR antibody (1:1000, ab101678, Abcam), anti‐A3R antibody (1:1000, ab197350, Abcam), anti‐A2BR antibody (1:1000, 27,987, Cell Signaling), anti‐AMPK antibody (1:1000, 2535, Cell Signaling), anti‐p‐AMPK antibody (1:1000, 2537, Cell Signaling), and anti‐GAPDH antibody (1:1000, AF4718, Affinity).

### 2.13 Metabolomic Analysis

Plasma samples of mice in each group were collected; the supernatant of plasma was obtained by centrifugation and stored in the refrigerator at −80°C. Metabolomic analysis of serum was performed by liquid chromatography combined with quadrupole time‐of‐flight mass spectrometry (UHPLC‐Q‐TOF/MS).

The serum (100 μL) was added with a mixture of acetonitrile and methanol (acetonitrile: methanol = 1:1) supplemented with 0.02 mg/mL L‐2‐chlorophenylalanine for extraction. After vortexing (30 s), ultrasonic extraction (5°C, 40 KHz), and centrifugation (4°C, 13,000 *g*, 15 min), the supernatant liquid was retained for the fluid under test.

The fluid under test (3 μL) was separated in an HSS T3 chromatographic column (100 mm × 2.1 mm i.d., 1.8 μm). The mobile phases A were 95% water and 5% acetonitrile (with 0.1% formic acid); the mobile phases B were 47.5% acetonitrile + 47.5% isopropanol + 5% water (with 0.1% formic acid). The column temperature was 40°C, and the flow rate was 0.40 mL/min. The MS signal adopted the positive and negative ion scanning mode. MS conditions were as follows: the mass scanning range was 70–1050 m/z; positive ion voltage was 3500; negative ion voltage was 2800 V; sheath gas was 40 psi; auxiliary heating gas was 10 psi; ion source heating temperature was 400°C; cyclic collision energy was 20‐40‐60 V; MS1 resolution was 70,000; MS^2^ resolution was 17,500.

### 2.14 Bioinformatics Analysis

Limma and VennDiagram packages in R software were utilized to identify the differential metabolites among the five groups: sham, model, low‐dose YQJPXY, middle‐dose YQJPXY, high‐dose YQJPXY. The selection thresholds were set as *p* < 0.05 and |log_2_ fold change (FC)| > 0.1. Partial Least‐Squares Discriminant Analysis (PLS‐DA) was conducted to explore the classification effect of the important differential metabolites using the ropls package in R. MetaboAnalyst database was used to investigate functional pathways enriched in the differential metabolites. Furthermore, a metabolite‐metabolite interaction network was constructed to investigate the links among the differential metabolites and to select the most important metabolites.

### 2.15 LC/MS Detection

The content of adenosine monophosphate, hypoxanthine, adenine, adenosine, and inosine in serum was detected by an Agilent RRHD SB‐C18 column (1.8 μm, 2.1 × 100 mm). The mouse serum (2 mL) was centrifuged at 5000 r/min for 5 min, and the supernatant was filtrated using a 0.45 μm microporous membrane. The filtered liquid was tested on the machine as a test solution. The chromatographic separation was performed at a flow rate of 0.4 mL/min and a temperature of 30°C in a column chamber. The mobile phase A is a 0.02 mmol/L ammonium acetate aqueous solution, and the mobile phase B is methanol. Elution conditions were set to: 0.00s, 98 A, 2% B; 3.00, 98 A, 2% B; 3.01, 90 A, 10% B; 5.00, 90 A, 10% B; 5.01, 98 A, 2% B; 6.00, 98 A, and 2% B.

### 2.16 Statistical Analysis

Statistical analyses were conducted by GraphPad Prism 7.0 and R software 4.2.2. All data were exhibited as mean ± standard deviation. Student's *t*‐test was used for the comparison of differences between two groups; one‐way analysis of variance (ANOVA) with Tukey's post hoc test was applied for comparison of differences among multiple groups. *p* < 0.05 was set as statistical significance.

## 3 Results

### 3.1 YQJPXY Attenuates Kidney Fibrosis in the Kidney Fibrosis Mouse Model

**FIGURE 1:**
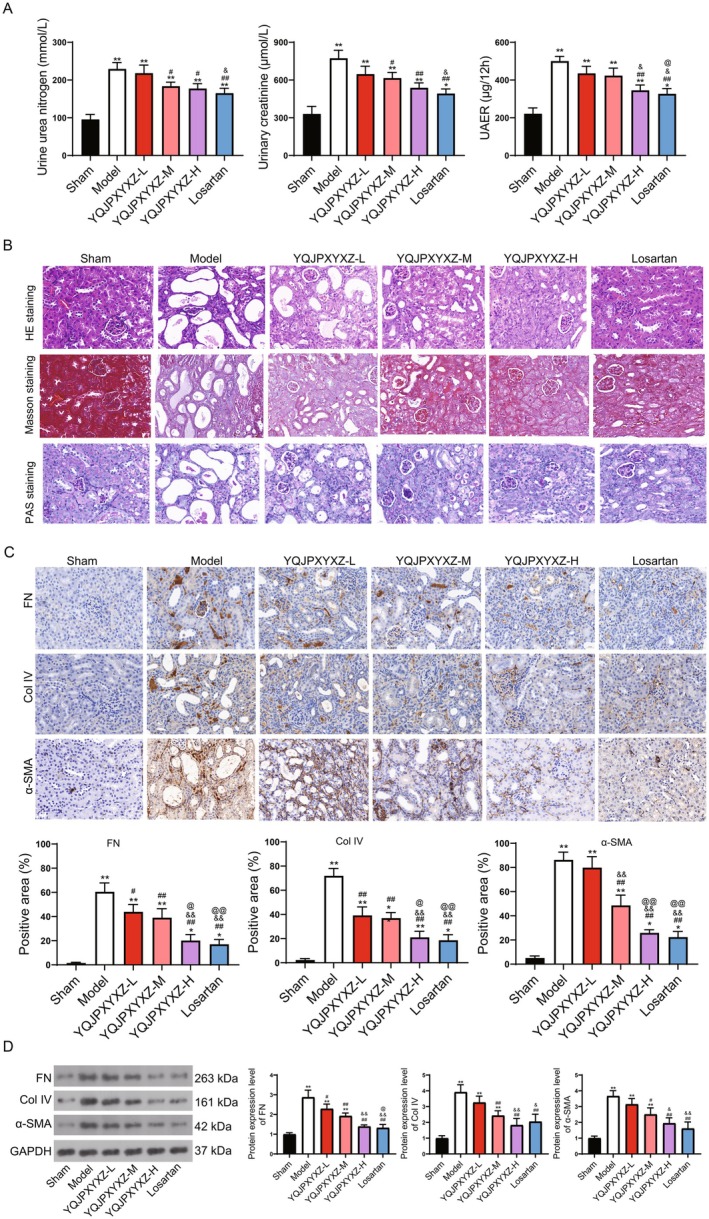
YQJPXY attenuates kidney fibrosis in kidney fibrosis mouse model. (A) UN, UCR, and UAER concentrations. (B) HE, Masson, and PAS staining for kidney tissues. Scale bar = 20 μm. (C) Immunohistochemical for detection of fibrosis markers FN, Col IV, and α‐SMA. Scale bar = 20 μm. (D) WB for detection of fibrosis markers FN, Col IV, and α‐SMA. YQJPXY‐L, low‐dose YQJPXY; YQJPXY‐M, middle‐dose YQJPXY; YQJPXY‐H, high‐dose YQJPXY. Compared to sham group, *p < 0.05, **p < 0.01; Compared to model group, #*p* < 0.05, ##*p* < 0.01; Compared to low‐dose YQJPXY, &*p* < 0.05, &&*p* < 0.01; Compared to middle‐dose YQJPXY, @*p* < 0.05, @*p* < 0.01.

UUO was used to establish the kidney fibrosis mouse model to investigate the effects of YQJPXY on kidney fibrosis. Significantly, the UN, UCR, and UAER were increased in the model groups, whereas they were further decreased in the YQJPXY and Losartan treatments (Figure 1A), indicating that YQJPXY attenuated kidney function. HE staining suggested no abnormality in renal tissue structure in the sham group, whereas renal tubular epithelial cells in the model group were atrophied, the lumen dilated with varying sizes, interstitial edema and widening were obvious, and inflammatory cells increased. The abnormality in the model group was alleviated after YQJPXY and Losartan administrations (Figure 1B). Masson staining found similar results: the renal structure of the sham group was normal, with only a few linear distributions of collagen fibers in the basal membrane of the glomerulus, mesangial region, and around the tubules. In contrast, in the model group, renal tubules atrophied significantly, and a large number of collagen fibers were observed. The fibrosis degree in the YQJPXY and Losartan groups was significantly improved compared to that in the model group (Figure 1B). PAS staining also indicated that tubule basement membranes were thickened, and renal tubules were multifocally atrophied in the model groups, whereas these abnormal changes were reversed in the YQJPXY and Losartan treatments (Figure 1B). Furthermore, the immunohistochemistry and WB results indicated that the fibrosis markers FN, Col IV, and α‐SMA also declined after YQJPXY treatment (Figure 1C,D). These findings indicate the alleviating effects of YQJPXY on kidney fibrosis.

### 3.2 Identification of the Differential Metabolites in Kidney Fibrosis

**FIGURE 2:**
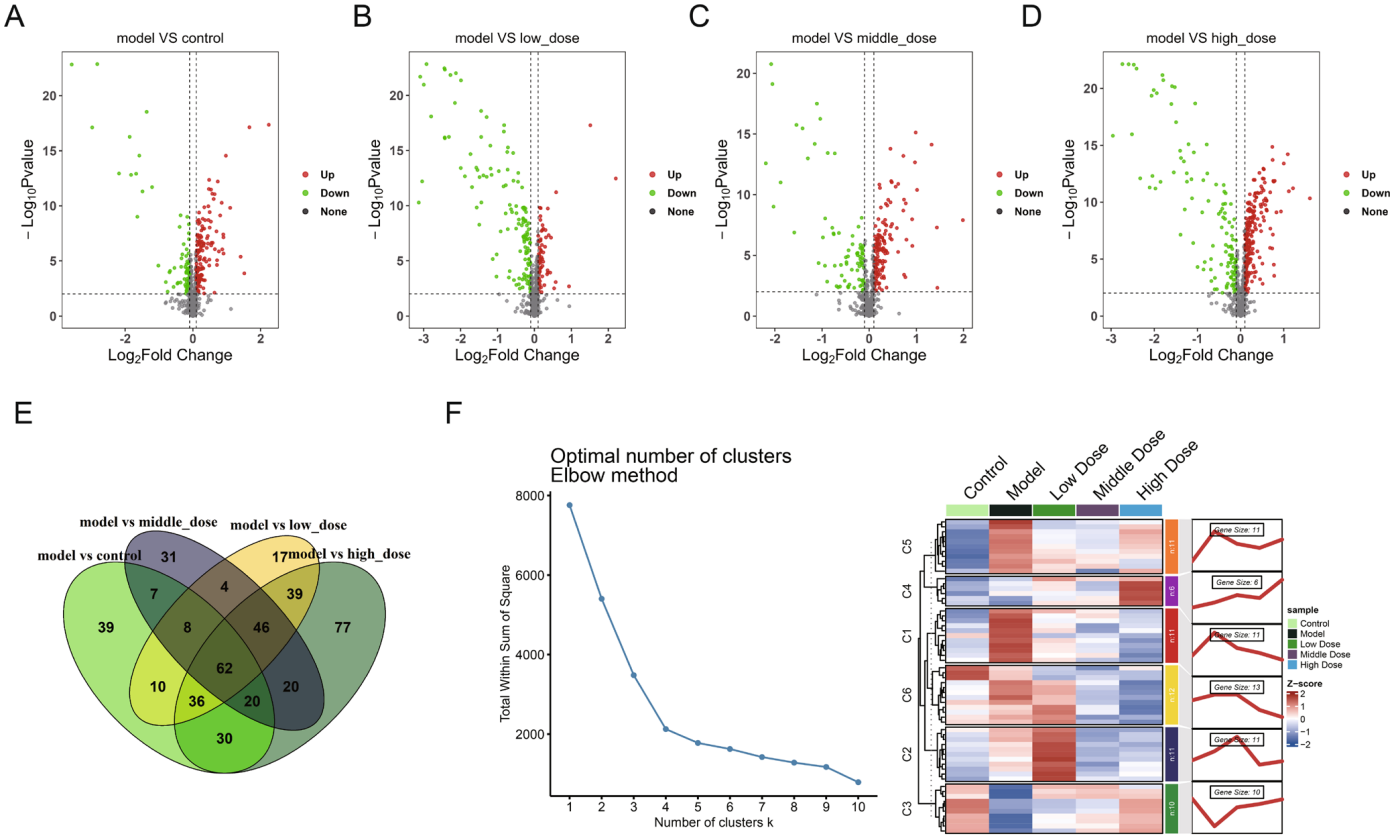
Identification of the differential metabolites in kidney fibrosis. (A) Volcano plot shows the differential metabolites between sham and model groups. (B) Volcano plot shows the differential metabolites between model and low‐dose YQJPXY groups. (C) Volcano plot shows the differential metabolites between model and middle‐dose YQJPXY groups. (D) Volcano plot shows the differential metabolites between model and high‐dose YQJPXY groups. (E) Venn analysis for investigation of important metabolites. (F) Trend cluster analysis.

To investigate the important metabolites in kidney fibrosis, non‐targeted metabolomics analyses were conducted using the blood samples from mice. Through differential expression analysis, 212 differential metabolites were identified between the sham and model groups, with 141 up‐regulated and 71 down‐regulated in the model group (Figure 2A). Between the model and low‐dose groups, 198 differential metabolites were identified, with 72 up‐ and 126 down‐regulated in the model group (Figure 2B). Between the model and middle‐dose groups, 222 differential metabolites were identified, with 141 up‐ and 81 down‐regulated in the model group (Figure 2C). Between the model and high‐dose groups, 330 differential metabolites were identified, with 215 up‐ and 115 down‐regulated in the model group (Figure 2D). Venn analysis indicated that 62 metabolites were intersected in the above four pairs of comparison groups (Figure 2E). To further select more significant metabolites in the process of YQJPXY against kidney fibrosis, trend cluster analysis was performed on the 62 metabolites, and metabolites that remained up‐down‐up‐regulated (or down‐up‐down‐regulated) between the three groups (normal–model–YQJPXY treatment) were screened for subsequent analysis. A total of 32 candidate metabolites were identified (Figure 2F).

### 3.3 Functional Analysis for the Metabolites

**FIGURE 3:**
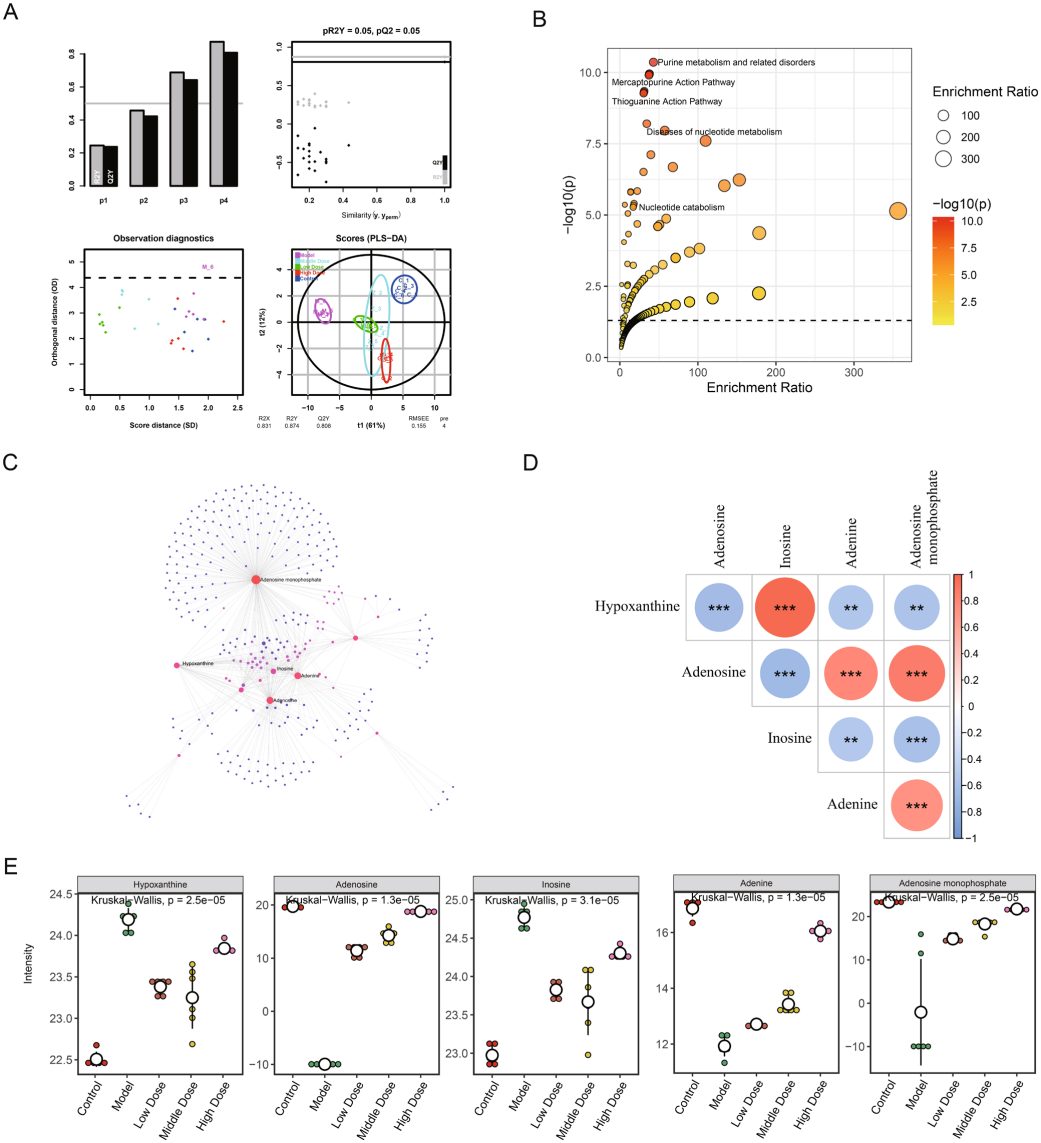
Identification of the differential metabolites in kidney fibrosis. (A) Partial Least‐Squares Discriminant Analysis (PLS‐DA) scatter plot shows the classification of the 62 differential metabolites. (B) Scatter plot of the pathways. The smaller the *p*‐value, the closer the color is to red. The size of the point represents impact. (C) Metabolite‐metabolite interaction networks. (D) Correlation analysis for the metabolites. (E) The levels of the five key metabolites in different groups.

To explore the important roles of the 32 metabolites in kidney fibrosis, PLS‐DA and functional analysis were conducted. The PLS‐DA results found that on the basis of the 32 candidate metabolites, all the samples were distinctly divided into five groups, which were consistent with our experimental groups (Figure 3A). This result further implied the important classified role of the 32 metabolites in the identification of disease, normal, and drug‐given groups. To further investigate the biological functions involved in the 32 metabolites, pathway enrichment analysis was performed. The results indicated that the 32 metabolites were correlated with 505 metabolism‐ and lipid‐related pathways, among which 301 were significantly enriched (Figure 3B). The significantly enriched pathways included purine metabolism and related disorders, the mercaptopurine action pathway, the thioguanine action pathway, and nucleotide catabolism.

### 3.4 Identification of the Key Metabolites in Kidney Fibrosis

To investigate the links among the 32 metabolites, a metabolite‐metabolite interaction network was constructed, and 667 metabolite‐metabolite interaction pairs were identified (Figure 3C). Among all the 32 metabolites, five metabolites were considered the optimal metabolites with the largest degree in the metabolite‐metabolite interaction network: Adenosine monophosphate (C00020), Adenosine (C00212), Adenine (C00147), Inosine (C00294), and Hypoxanthine (C00262). Correlation analysis implied that the five metabolites were significantly related to each other (Figure 3D). The content of these metabolites among different experimental groups was also investigated. The results showed that Hypoxanthine and Inosine were significantly increased in the model groups when compared with the control, but were significantly decreased in the YQJPXY given groups. Adenine, Adenosine monophosphate, and Adenosine were significantly down‐regulated in the model group, whereas they were significantly up‐regulated in the medicine groups (Figure 3E).

### 3.5 Adenosine Attenuates Kidney Fibrosis in the Kidney Fibrosis Mouse Model

**FIGURE 4:**
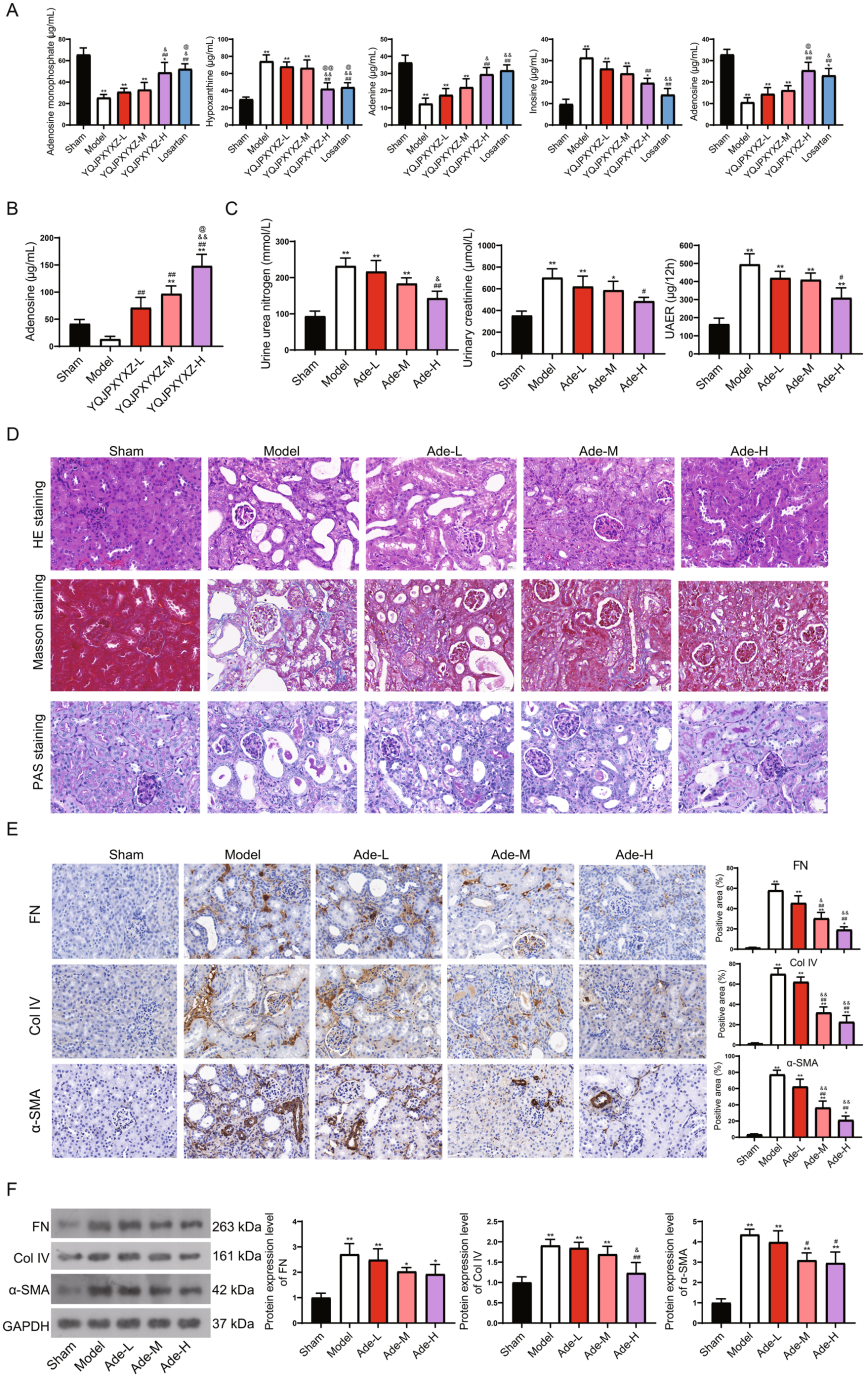
Adenosine attenuates kidney fibrosis in a kidney fibrosis mouse model. (A) The concentrations of the five metabolites in different groups. (B) UN, UCR, and UAER concentrations. (C) Adenosine levels in different groups. (D) HE, Masson, and PAS staining for kidney tissues. Scale bar = 20 μm. (E) Immunohistochemical detection of fibrosis markers FN, Col IV, and α‐SMA. Scale bar = 20 μm. (F) WB for detection of fibrosis markers FN, Col IV, and α‐SMA. Compared to the sham group, *p < 0.05, **p < 0.01; Compared to the model group, #*p* < 0.05, ##*p* < 0.01; Compared to low‐dose YQJPXY, &*p* < 0.05, &&*p* < 0.01; Compared to middle‐dose YQJPXY, @*p* < 0.05, @@*p* < 0.01.

To confirm the content alteration of the five key metabolites in kidney fibrosis, we detected the concentration of Adenosine monophosphate, Adenosine, Adenine, Inosine, and Hypoxanthine in various mice groups using targeted LC/MS. The results showed that Adenosine monophosphate, Adenine, and Adenosine significantly declined in the model group, whereas they were significantly increased after YQJPXY administration. Hypoxanthine and Inosine were significantly elevated in the model group, whereas they significantly declined after YQJPXY administration (Figure 4A). These results were similar to the non‐targeted prediction.

Adenosine is an organic compound belonging to the purine nucleoside group, which exhibited a protective role in kidney diseases (Pandey et al. 2021). To investigate the specific roles of Adenosine in kidney fibrosis, different concentrations of Adenosine were used to treat the kidney fibrosis mouse model. After Adenosine treatment, the Adenosine levels in the mouse serum were significantly increased in a dose‐dependent manner (Figure 4B). Adenosine addition markedly improved renal function (Figure 4C) and kidney fibrosis (Figure 4D–F). To be specific, the increased UN, UCR, and UAER levels in the model groups were significantly decreased in the Adenosine addition groups, indicating improved kidney function (Figure 4C); the atrophic renal tubular epithelial cells, dilated lumen, increased inflammatory cells, and increased fibrosis degree in the model groups were remised by Adenosine addition (Figure 4D); the fibrosis proteins FN, Col IV, and α‐SMA in the model group also declined in the Adenosine addition groups (Figure 4E,F). This evidence indicated that Adenosine was beneficial for renal function recovery and fibrosis alleviation.

### 3.6 Adenosine Promotes Autophagy in Kidney Fibrosis

Although the important roles of Adenosine had been demonstrated to be critical in the processes of YQJPXY against kidney fibrosis, the specific roles remained unclear. Autophagy has been shown to be closely related to the pathogenesis of various kidney diseases, including kidney fibrosis (Tang et al. 2020). The stimulation of Adenosine receptor A2 (A2BR) has been reported to activate adenylate cyclase and cyclic adenosine monophosphate (cAMP) (Sachdeva and Gupta 2013), which results in adenosine 5‘‐monophosphate (AMP)‐activated protein kinase (AMPK)‐induced autophagy (Afshari et al. 2023a, 2023b). Hence, we speculated that Adenosine attenuates kidney fibrosis through A2BR/cAMP/AMPK pathways in a kidney fibrosis mouse model.

**FIGURE 5:**
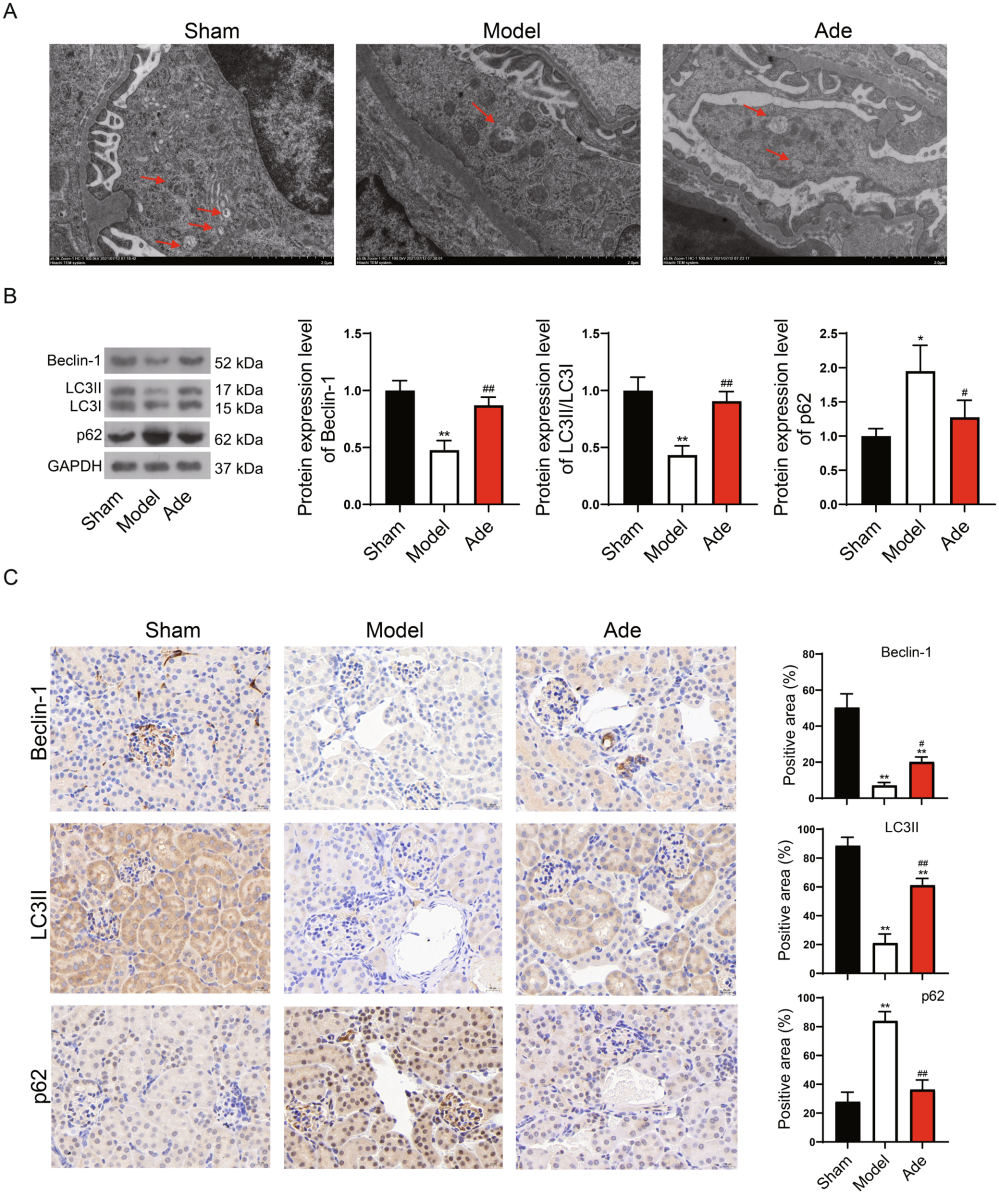
Adenosine promotes autophagy in kidney fibrosis. (A) TEM for observation of autophagic lysosomes. Red arrows represent the lysosomes. (B) WB for detection of autophagy markers Beclin‐1, LC3II/LC3I, and p62. (C) Immunohistochemical for detection of autophagy markers Beclin‐1, LC3II/LC3I, and p62. Ade, Adenosine addition group. Compared to sham group, *p < 0.05, **p < 0.01; Compared to model group, #*p* < 0.05, ##*p* < 0.01.

Firstly, the activation of autophagy induced by Adenosine was confirmed in the animal experiments. The TEM indicated that the number of autophagic lysosomes in the kidney tissue of the mouse model was less than that of the control, but the number of autophagic lysosomes in the Adenosine addition group was significantly increased (Figure 5A). The autophagy proteins Beclin‐1 and LC3II/LC3I were significantly increased by Adenosine addition, whereas p62 was significantly decreased by Adenosine addition (Figure 5B,C).

**FIGURE 6:**
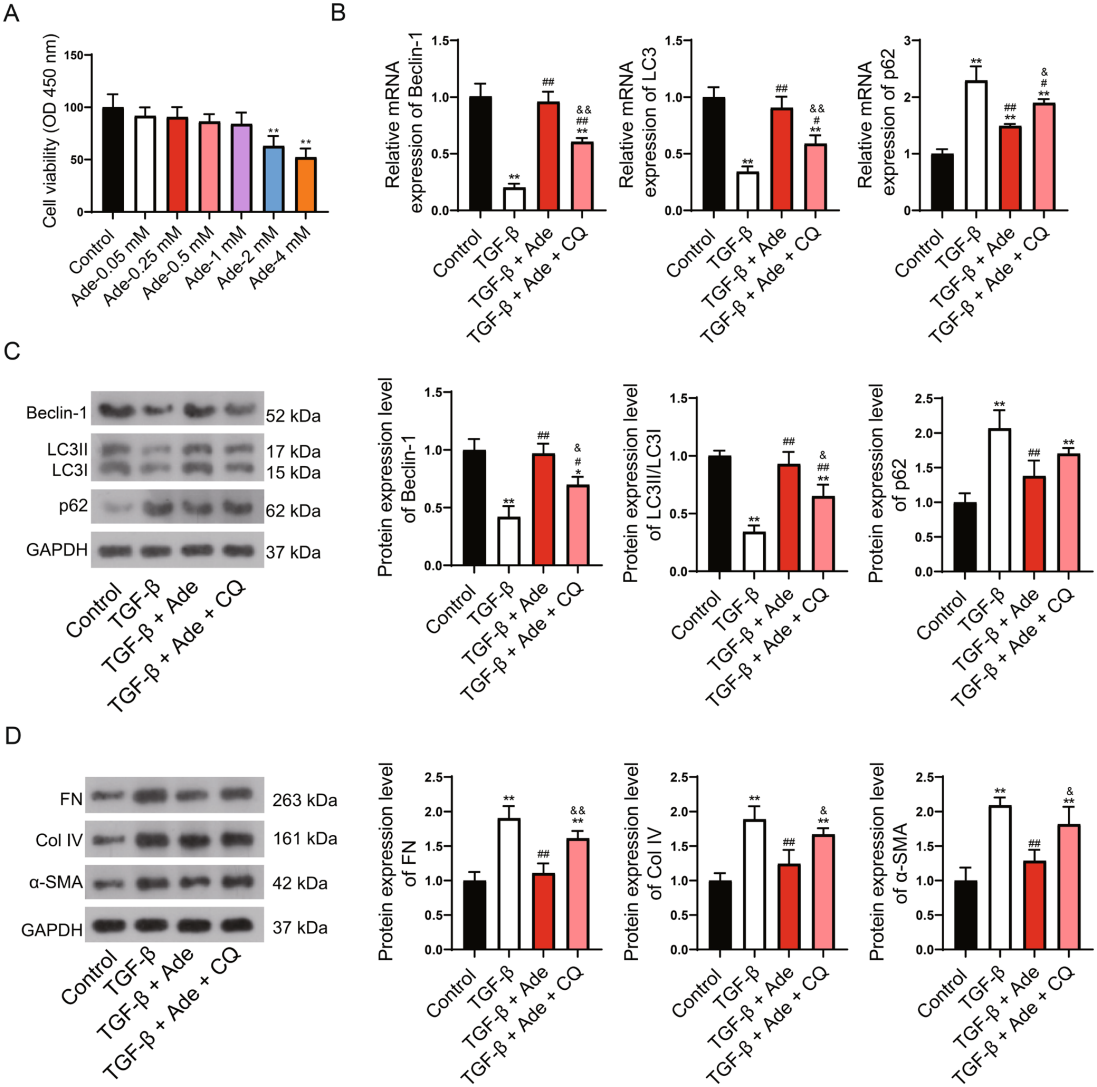
Adenosine promotes autophagy to attenuate kidney fibrosis. (A) The cell viability detected by CCK‐8. (B) qRT‐PCR for detection of autophagy markers Beclin‐1, LC3II/LC3I, and p62. (C) WB for detection of autophagy markers Beclin‐1, LC3II/LC3I, and p62. (D) WB for detection of fibrosis markers FN, Col IV, and α‐SMA. Compared to control, *p < 0.05, **p < 0.01; Compared to TGF‐β, #*p* < 0.05, ##*p* < 0.01; Compared to TGF‐β + Ade, &*p* < 0.05, &&*p* < 0.01.

Secondly, the cell experiments were carried out using the kidney cell line HK‐2. In a separate set of experiments, we found that moderate Adenosine (less than 2 mM) was safe for HK‐2 cell viability, and excess adenosine (above 2 mM) caused HK‐2 damage (Figure 6A). Hence, 1 mM was selected to be the treatment concentration of Adenosine for HK‐2 cells in the subsequent experiments. TGF‐β was used to construct a kidney fibrosis cell model, and chloroquine (CQ) was used for autophagy inhibition. qRT‐PCR and WB indicated that the expression levels of Beclin‐1 and LC3II/LC3I were decreased, and p62 was increased in the TGF‐β‐induced fibrosis cell model, whereas these trends were reversed by Adenosine (Figure 6B,C). Similarly, the increased fibrosis biomarkers FN, Col IV, and α‐SMA induced by TGF‐β were also inhibited by Adenosine (Figure 6D). This evidence indicated that Adenosine promoted autophagy to attenuate kidney fibrosis, which was consistent with the results in animal experiments. Interestingly, the activation of autophagy and the decreased fibrosis under Adenosine addition were further inhibited by CQ (Figure 6C,D), indicating that Adenosine attenuated kidney fibrosis through activation of autophagy.

### 3.7 Adenosine Promotes Autophagy to Attenuate Kidney Fibrosis Through A2BR/cAMP/AMPK Pathway

**FIGURE 7:**
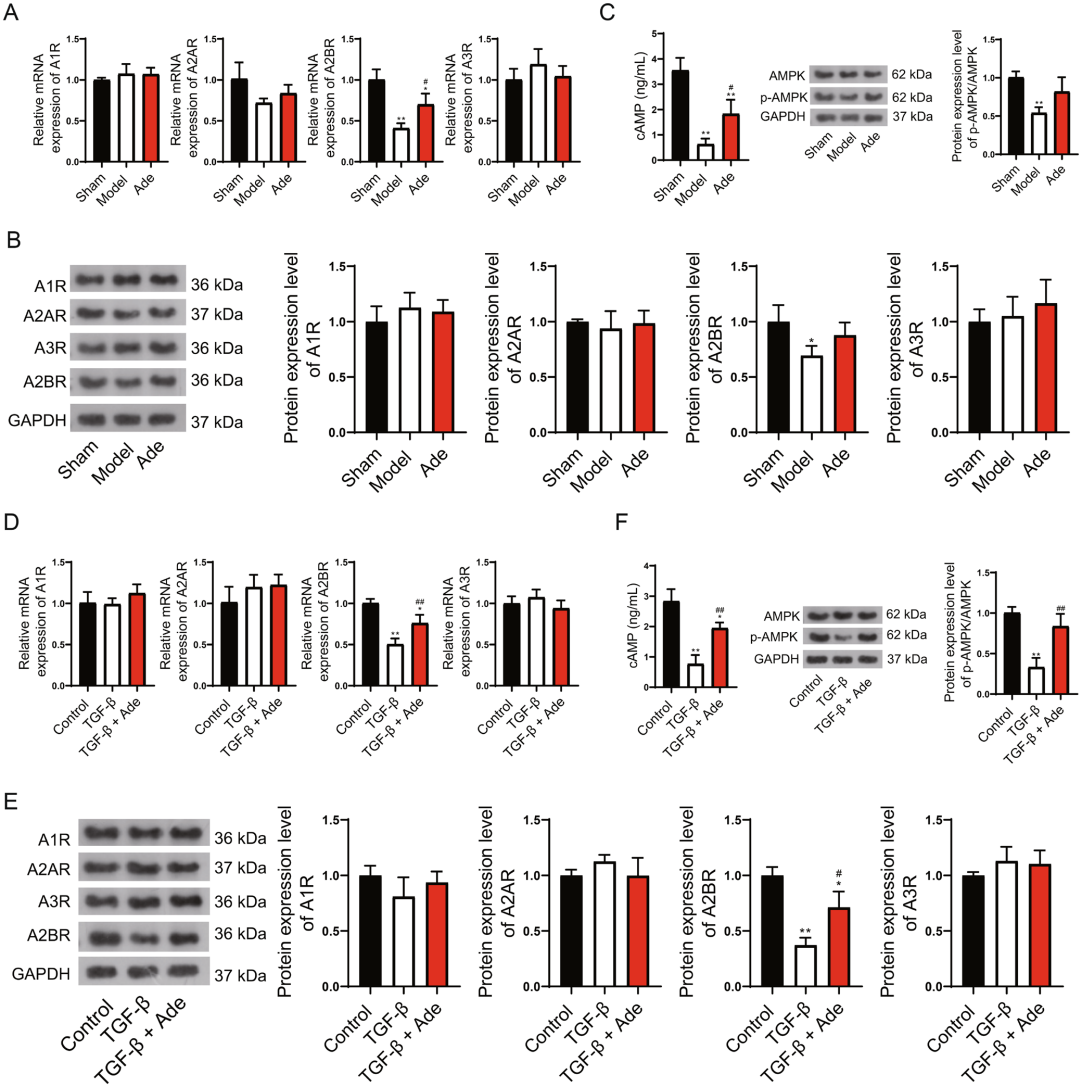
Adenosine promotes autophagy to attenuate kidney fibrosis through the A2BR/cAMP/AMPK pathway. (A) qRT‐PCR for detection of Adenosine receptors (A1R, A2AR, A2BR, and A3R). (B) WB for detection of Adenosine receptors (A1R, A2AR, A2BR, and A3R). (C) ELISA for detection of cAMP and WB for detection of AMPK and p‐AMPK. (D) qRT‐PCR for detection of Adenosine receptors (A1R, A2AR, A2BR, and A3R). (E) WB for detection of Adenosine receptors (A1R, A2AR, A2BR, and A3R). (F) ELISA for detection of cAMP and WB for detection of AMPK and p‐AMPK. Compared to control, *p < 0.05, **p < 0.01; compared to TGF‐β, #*p* < 0.05, ##*p* < 0.01.

To confirm that the occurrence of autophagy was induced by the A2BR/cAMP/AMPK pathway in the process of Adenosine against kidney fibrosis, animal and cell experiments were performed. In the animal studies, A2BR was reduced in the model group and was elevated in the Adenosine given group, although this increase was not significant (Figure 7A,B). Furthermore, the cAMP and p‐AMPK/AMPK levels were reduced in the model group and were next elevated in the Adenosine given group in the kidney tissues (Figure 7C). Similar results were also observed in the cell experiments, indicating up‐regulated A2BR, cAMP, and p‐AMPK/AMPK in the Adenosine addition groups (Figure 7D–F).

**FIGURE 8:**
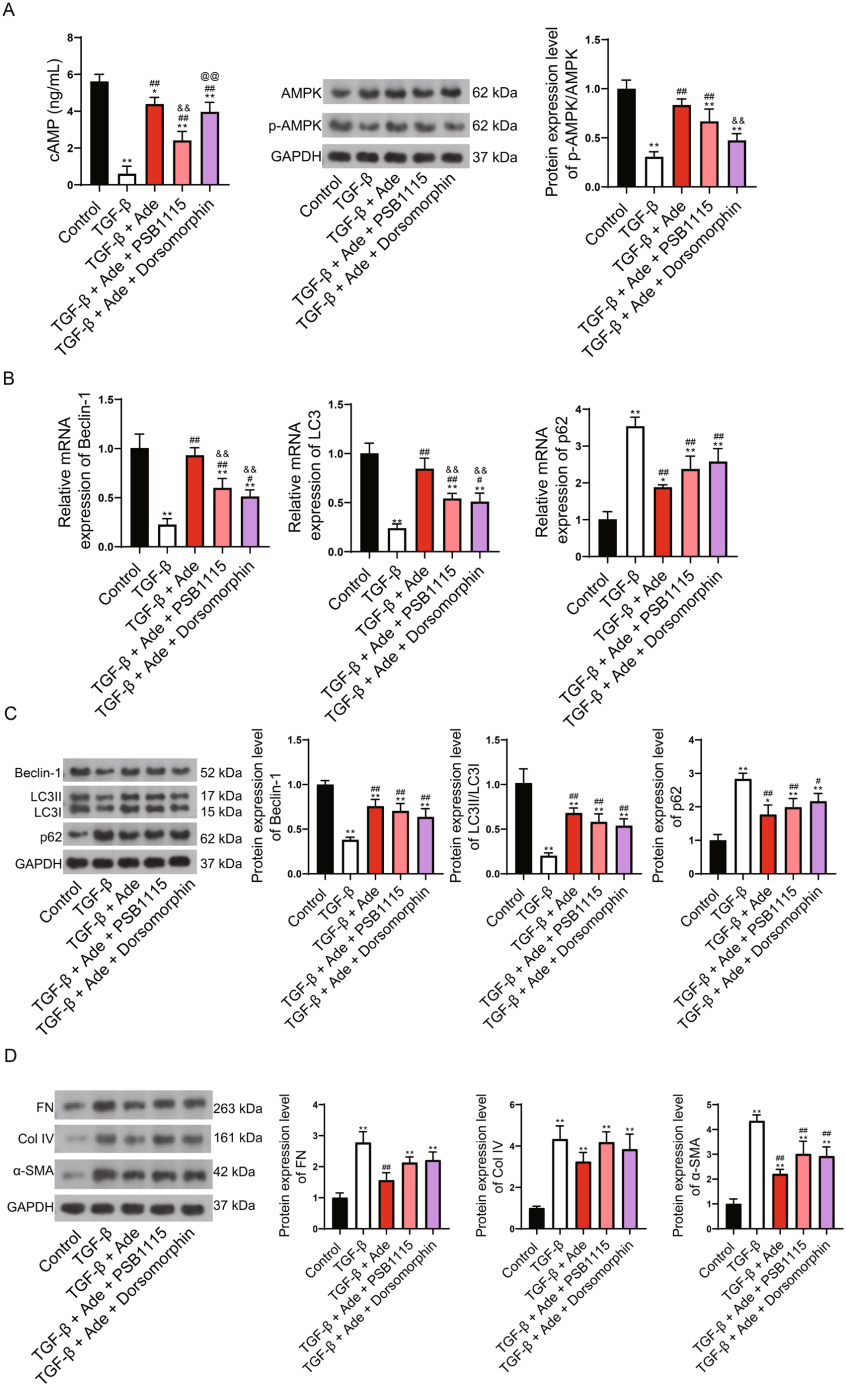
Adenosine promotes autophagy to attenuate kidney fibrosis through the A2BR/cAMP/AMPK pathway. (A) ELISA for detection of cAMP and WB for detection of AMPK and p‐AMPK. (B) qRT‐PCR for detection of autophagy markers Beclin‐1, LC3II/LC3I, and p62. (C) WB for detection of autophagy markers Beclin‐1, LC3II/LC3I, and p62. (D) WB for detection of fibrosis markers FN, Col IV, and α‐SMA. Compared to control, *p < 0.05, **p < 0.01; Compared to TGF‐β, #*p* < 0.05, ##*p* < 0.01; Compared to TGF‐β + Ade, &*p* < 0.05, &&*p* < 0.01; Compared to TGF‐β + Ade + PSB1115, @*p* < 0.05, @@*p* < 0.01.

Following, A2BR inhibitor PSB1115 and AMPK inhibitor Dorsomorphin were added to the TGF‐β‐induced fibrosis model. The results implied that the increased cAMP, p‐AMPK/AMPK, Beclin‐1, LC3II/LC3I, decreased p62, and increased fibrosis biomarkers (FN, Col IV, and α‐SMA) induced by Adenosine addition were reversed by PSB1115 and Dorsomorphin (Figure 8). This evidence indicated that Adenosine promoted autophagy to attenuate kidney fibrosis through the A2BR/cAMP/AMPK pathway.

## 4 Discussion

YQJPXY exhibits efficacy in promoting blood circulation, removing blood stasis, and clearing away turbid toxins (Ji et al. 2024). In our study, the renal function recovery and antifibrotic effect of YQJPXY were confirmed in the UUO mouse model, manifesting as decreased UN, UC, UAER, kidney tissue injury, and kidney fibrosis. Metabolomic analysis identified 62 significantly different metabolites that were dysregulated under YQJPXY treatment. Metabolite‐metabolite interaction network and functional analysis found five important metabolites, which might act as effective mediators in fibrosis in kidney disease: Adenosine monophosphate, Adenosine, Adenine, Inosine, and Hypoxanthine.

Adenine can increase serum creatinine, decrease creatinine clearance, and produce proteinuria, which is the main symptom of renal dysfunction; and induce kidney fibrosis, like increased α‐SMA, FSP‐1, MMP‐2, and decreased cytokeratin (Diwan et al. 2018; Haritha et al. 2022). The Adenine addition‐induced kidney fibrosis model has been widely used for the exploration of various mechanisms in kidney disease and related cardiovascular disease (Haritha et al. 2022). In our study, Adenine level decreased in kidney fibrosis mice and increased after YQJPXY treatment, further demonstrating the crucial roles of Adenine in the process of kidney fibrosis. Furthermore, Hypoxanthine has also been demonstrated to be highly correlated with kidney fibrosis (Ohtsubo et al. 2009). Hypoxanthine has been widely used for the construction of a hyperuricemia mouse model, and the accumulation of Hypoxanthine can induce kidney fibrosis (Zeng et al. 2024). Some TCMs like Polygonati Rhizoma polysaccharide (Zhang et al. 2024) and Fuling‐Zexie formula (Lu et al. 2024) are reported to exhibit anti‐fibrosis effects in kidney diseases through lowering the Hypoxanthine‐induced hyperuricemia, which were coincident with our results. Inosine levels were significantly elevated in the UUO model and were significantly decreased by YQJPXY administration. Inosine inhibitor has also been used for fibrosis blocking (Herman‐de‐Sousa et al. 2020; Nakanishi et al. 2010), which is also coincident with our results. Adenosine monophosphate (AMP) is involved in glucose and lipid metabolism. In diabetic nephropathy, AMPK plays a pivotal role in cell growth and cellular energy homeostasis, and the dysregulation of AMPK is a pivotal component of the development of metabolic syndrome and type 2 diabetes mellitus. AMPK activation improves glucose and lipid homeostasis in insulin‐resistant animal models (Kim and Park 2016). Some studies have indicated the regulatory role of AMP in kidney fibrosis progression and therapy process. For example, metformin protects tubular cells from inflammation and oxidative stress through activation of AMPK and thereby improves kidney fibrosis (Song et al. 2021). Berberine and Yishen Huoxue decoction have also been demonstrated to exert amelioration roles in renal interstitial inflammation and fibrosis, which largely rely on the AMPK activation (Tan et al. 2023; Zhong et al. 2021). However, the specific mechanism of AMP under the administration of YQJPXY in the kidney remains unclear. Previously published reports indicate that YQJPXY can mediate the PI3K/Akt/mTOR signaling pathway to improve kidney fibrosis in DN mice (F. Chen et al. 2021), and inhibition of PI3K/Akt/mTOR is related to activation of the AMPK pathway (Chen et al. 2022); hence, we speculated that the mechanism of YQJPXY relieving kidney fibrosis might be mediated by AMP. Activation of Adenosine receptors A2A and A2B has been demonstrated to reduce inflammatory response before kidney fibrosis (Roberts et al. 2014). N6‐(2‐Hydroxyethyl) adenosine from Cordyceps cicadae is reported to improve renal interstitial fibrosis and inhibit inflammation through the regulation of TGF‐β1/Smad and NF‐κB pathways (Zheng et al. 2018). In our results, Adenosine dysfunction is also demonstrated; hence, we speculated that YQJPXY might also relieve kidney fibrosis through regulating Adenosine‐related biological functions and pathways. Collectively, the five metabolites identified in our analysis might all be important in the processes of YQJPXY against kidney fibrosis.

Cause that the specific mediatory mechanism of Adenosine monophosphate, Adenine, Inosine, and Hypoxanthine has been investigated in various publications, we selected Adenosine as the most important metabolite to investigate its underlying mechanisms in the process of YQJPXY against kidney fibrosis. In both animals and cells, Adenosine addition significantly improved kidney function and inhibited fibrosis in our results. Adenosine is reported to be an endogenous purine nucleoside and can mediate different biological effects through purinergic receptors (Sachdeva and Gupta 2013). At present, four established Adenosine receptors have been cloned, which are A1R, A2AR, A2BR, and A3R. In our results, alteration of A2AR was significant with the Adenosine addition. It is reported that the stimulation of A2BR activates the adenylate cyclase and cAMP (Sachdeva and Gupta 2013), which results in the AMPK‐induced autophagy (Afshari et al. 2023a, 2023b; Lan et al. 2022; Rueda et al. 2021; Tang et al. 2023; Zhou et al. 2017). Hence, we speculated that Adenosine attenuates kidney fibrosis through A2BR/cAMP/AMPK pathways‐mediated autophagy in a kidney fibrosis mouse model. Autophagy, as an important stress response system, has been shown to be involved in the pathogenesis of various kidney diseases, including kidney fibrosis (Livingston et al. 2023, 2024; Wang et al. 2024). In our results, the number of autophagic lysosomes was increased by Adenosine addition, with the increased autophagy biomarkers Beclin‐1 and LC3II/LC3I, and decreased biomarker p62, which indicates the activation of autophagy in kidney tissues and cells. When the autophagy inhibitor QC was added, activated autophagy and inhibited fibrosis induced by Adenosine addition were blocked, indicating that the Adenosine addition exerted its therapeutic and antifibrotic roles through activation of autophagy. Moreover, with the addition of A2BR and cAMP/AMPK pathway inhibitors, the declined fibrosis and activated autophagy, accompanied by Adenosine addition, were also reversed, indicating that Adenosine can activate autophagy to attenuate kidney fibrosis through A2BR/cAMP/AMPK pathways in kidney fibrosis.

Our study emphasized the principal role of Adenosine through regulating the A2BR/cAMP/AMPK pathway‐induced autophagy in YQJPXY‐treated kidney fibrosis. Nevertheless, there are still some limitations. First, although the protective role and the specific mechanism of Adenosine in the treatment of YQJPXY against kidney fibrosis have been demonstrated in the present observation, this is largely based on animal and cell experiments. Second, the regulatory role of Adenosine monophosphate, Adenine, Inosine, and Hypoxanthine is absent in our investigations, and more validations are necessary in the future. Finally, the clinical application of these metabolites also needs to be explored to benefit disease management and treatment.

## 5 Conclusion

Through kidney fibrosis mouse model construction and metabolomic analysis, YQJPXY significantly alleviated fibrosis by regulating the expression of five principal metabolites: Adenosine monophosphate, Adenosine, Adenine, Inosine, and Hypoxanthine in serum. Both in metabolomic analysis and LC/MS detection, Adenine, Adenosine monophosphate, Adenosine, and Adenine were significantly down‐regulated in the model group, whereas Hypoxanthine and Inosine were significantly up‐regulated in the YQJPXY groups. Furthermore, Adenosine is demonstrated to activate autophagy to attenuate kidney fibrosis through A2BR/cAMP/AMPK pathways in kidney fibrosis. Our findings revealed the antifibrotic mechanism of YQJPXY against kidney fibrosis and provided the experimental basis for clinical application.

## Author Contributions

**Keda Lu:** funding acquisition (equal), writing – original draft (equal). **Liqing Ye:** writing – original draft (equal). **Wenze Jiang:** data curation (equal), software (equal). **Tianyang Cheng:** data curation (equal), software (equal). **Hong Xia:** data curation (equal), software (equal). **Peipei Zhang:** writing – review and editing (equal). **Bingbing Zhang:** writing – review and editing (equal).

## Ethics Statement

The animal study was reviewed and approved by the Institutional Animal Care and Use Committee of Zhejiang Chinese Medical University (Approval No. IACUC‐20221219‐12), which conformed to the National Institutes of Health Guidelines for the Care and Use of Laboratory Animals, and methods were reported in accordance with ARRIVE guidelines.

## Consent

The authors have nothing to report.

## Conflicts of Interest

The authors declare no conflicts of interest.

## Data Availability

The original datasets used during the current study are available from the corresponding author on reasonable request.
